# Larval fish body growth responses to simultaneous browning and warming

**DOI:** 10.1002/ece3.8194

**Published:** 2021-10-04

**Authors:** Magnus Huss, Renee M. van Dorst, Anna Gårdmark

**Affiliations:** ^1^ Department of Aquatic Resources Swedish University of Agricultural Sciences Öregrund Sweden; ^2^ Department of Biology and Ecology of Fishes Leibniz‐Institute of Freshwater Ecology and Inland Fisheries Berlin Germany

**Keywords:** Baltic Sea, climate change, perch, temperature, water color, zooplankton

## Abstract

Organisms are facing global climate change and other anthropogenic pressures, but most research on responses to such changes only considers effects of single drivers. Observational studies and physiological experiments suggest temperature increases will lead to faster growth of small fish. Whether this effect of warming holds in more natural food web settings with concurrent changes in other drivers, such as darkening water color (“browning”) is, however, unknown. Here, we set up a pelagic mesocosm experiment with large bags in the Baltic Sea archipelago, inoculated with larval Eurasian perch (*Perca fluviatilis*) and zooplankton prey and varying in temperature and color, to answer the question how simultaneous warming and browning of coastal food webs impact body growth and survival of larval perch. We found that browning decreased body growth and survival of larval perch, whereas warming increased body growth but had no effect on survival. Based on daily fish body growth estimates based on otolith microstructure analysis, and size composition and abundance of available prey, we explain how these results may come about through a combination of physiological responses to warming and lower foraging efficiency in brown waters. We conclude that larval fish responses to climate change thus may depend on the relative rate and extent of both warming and browning, as they may even cancel each other out.

## Graphical abstract

We experimentally studied the combined effects of increased temperature (“warming”) and darkening water color (“browning”) on larval fish body growth. Browning decreased body growth and survival of larval perch, whereas warming increased body growth but had no effect on survival. We conclude that larval fish responses to climate change thus may depend on the relative rate and extent of both warming and browning.

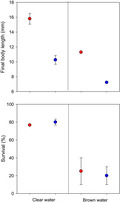

## 1 INTRODUCTION

With global climate change, many natural populations are forced to face novel combinations of environmental conditions. Studies of how climate change affects organisms, however, commonly focus on warming or other drivers alone, ignoring how they may act in combination (Boyd et al., 2018). Along with warming, many fresh‐ and coastal waters in the Northern Hemisphere have become browner due to increased concentrations of iron and dissolved organic matter as a result of increased run‐off from terrestrial watersheds (Creed et al., 2018; Solomon et al., 2015). The latter results from a combination of climate change, de‐acidification, and changes in land use (Kritzberg et al., 2020). Northern fish populations and food webs thus face and respond to simultaneous changes in water color and temperature.

Brown waters are correlated with lower fish body growth and production (Karlsson et al., 2015; Van Dorst et al., 2019). This can be explained in part by reduced (or altered source of) basal production through shading (Ask et al., 2009; Vasconcelos et al., 2016), whereas pelagic basal production responses vary from negative to positive (Kelly et al., 2018). In some cases, fish foraging rates are also lower in low‐light conditions (Weidel et al., 2017). Organisms’ responses (e.g., body growth) to warming, on the other hand, depend on changes in both individual metabolism and consumption rates (Brown et al., 2004; Englund et al., 2011). Because these rates also vary with body size, so can responses to warming (Gårdmark & Huss, 2020). For ectotherms, such as fish, warming‐induced changes in physiology therefore often increase growth or performance of small but not large individuals (Huss et al., 2019; Lindmark et al., 2018; Ohlberger, 2013), whereas browning can lead to lower body growth across fish life stages (Van Dorst et al., 2019). In a recent comparative lake study, Van Dorst et al. (2019) showed how browning and warming both may reduce total fish biomass production and still have differing effects on fish body growth.

To identify the causes of responses to variation in water color (Estlander et al., 2010), temperature (O'Gorman et al., 2016), and their combination (Van Dorst et al., 2019), as observed in observational studies, we need manipulative experiments. Earlier experiments have identified strong but varying responses in fish body growth to both warming (Handeland et al., 2008, Huss et al., 2019, see Gårdmark & Huss, 2020 for a more extensive list of studies on body growth responses to warming) and browning (Leech et al., 2021; Van Dorst et al., 2020). Responses may, for example, depend on body size due to temperature affecting the allometric scaling of metabolism and food intake (Lindmark et al., 2018) and light availability having different effects on prey resources preferred by small and large fish (Van Dorst et al., 2021; Vasconcelos et al., 2018). Still, we largely lack controlled experiments on fish body growth responses to simultaneous browning and warming, and with respect to browning on larval fish in general. In Hansson et al. (2013), however, they manipulated both temperature and light (by adding dissolved organic carbon, DOC) and found that both had a positive effect on body size of roach (*Rutilis rutilus*) larvae. This contrasts to findings in other experiments manipulating light availability (Van Dorst et al., 2020; Leech et al., 2021) and in comparative studies across lakes with different water color (Estlander et al., 2010; Van Dorst et al., 2019) that all show a negative effect of browning. This suggests that responses to browning are context‐dependent, depending on, for example, fish species and prey composition (Van Dorst et al., 2020) and the level and source of browning (e.g., with or without added nutrients, Finstad et al., 2014). Similarly, the extent to which warming has positive or negative effects on fish and other organisms depends on the species’ temperature optimum, life stage, and amount of warming relative the ambient temperature (Ohlberger, 2013).

Most research on responses to browning concerns organisms in freshwater ecosystems, and to the best of our knowledge, there is none concerning coastal fish. However, also coastal areas receive freshwater discharge with colored humic substances, resulting in reduced light penetration leading to shifts in coastal productivity (Wikner & Andersson, 2012). In many coastal regions in the Northern Hemisphere, river discharge and run‐off is predicted to increase with climate change due to increased frequency and intensity of rainfall (Straat et al., 2018). Shallow coastal waters, constituting key feeding and nursery grounds for many fish species (Kraufvelin et al., 2018), may be severely impacted if basal production controlled by light availability (Ask et al., 2016) is reduced by increased browning. This adds to a burden of other anthropogenic stressors (Reusch et al., 2018) including warming, which in northern coastal areas such as the Baltic Sea region is expected to exceed the global average (IPCC, 2014).

Here, we ask (1) how warmer and browner waters affect larval fish body growth and survival using the Eurasian perch (*Perca fluviatilis*), which is a common omnivorous fish species in freshwaters and coastal areas in the Baltic Sea region (Olsson, 2019), as our study species and (2) the extent to which those responses are mediated by shifts in the biomass of their zooplankton prey. To this end, we performed a fully factorial experiment of warming and browning in pelagic mesocosms in two adjacent areas in the Baltic Sea archipelago: an artificially heated coastal bay and a natural area with ambient temperatures. Browning was simulated by adding colored substances to the mesocosm bags, whereas the water masses surrounding them in the heated and the natural bay provided the temperature treatment. We find strong but contrasting effects of warming and browning on larval perch body growth and survival.

## 2 MATERIALS AND METHODS

### 2.1 Study species, site description, and experimental design

**FIGURE 1:**
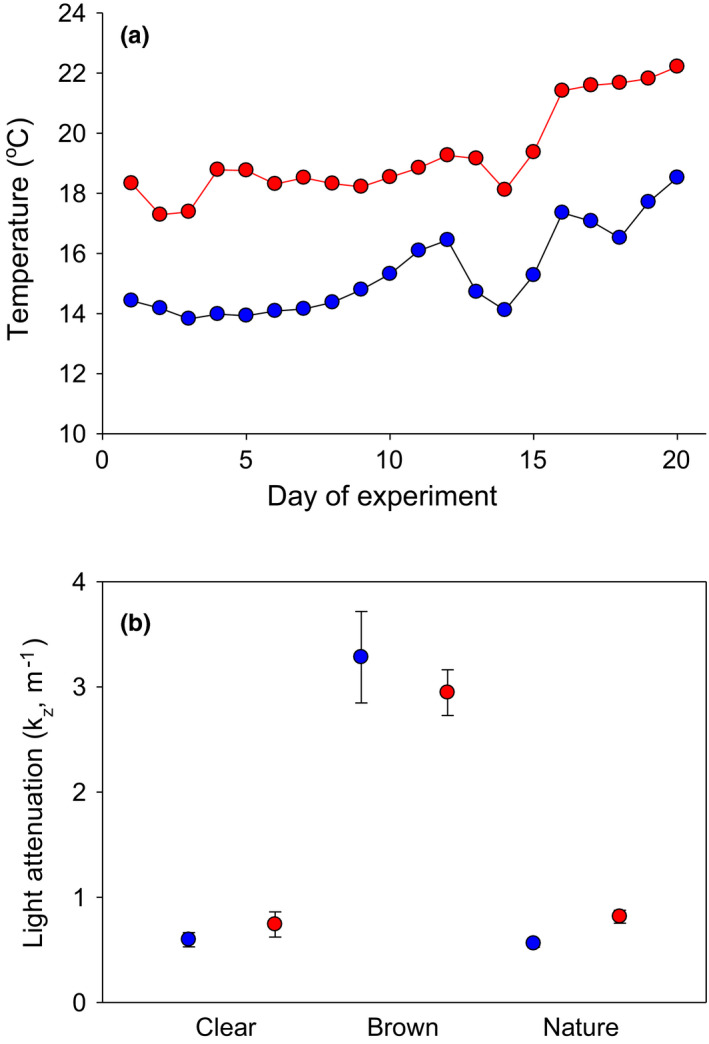
(a) Daily mean temperature measured at 1 m depth in the heated (red) and reference (blue) area and (b) light attenuation (mean ± SE) in different color treatments and areas at low (blue) or high (red) temperatures

We performed the mesocosm experiment in the Baltic Sea archipelago (60°42′N, 18°19′E with a salinity of about 4.5 psu) 31 May–19 June 2017. The focal species (Eurasian perch, hereafter “perch”) is common in the area (Adill et al., 2018) and across the coastal regions of the Baltic Sea (Olsson, 2019), as well as in lakes throughout the northern Eurasia (Johansson & Persson, 1986). Percids depend on vision for feeding (Helfman, 1979), and Eurasian perch accordingly feed less efficiently at low visibility (Estlander et al., 2012). We specifically designed this experiment to study responses of perch during their larval phase, which commonly lasts from hatching (~5–6 mm in length) until reaching a size of ~20 mm (Byström et al., 1998; Treasurer, 1988). We conducted the experiment in an enclosed coastal ecosystem (the Biotest Lake) of ~1 km^2^ in the Baltic Sea that receives heated cooling water from a nearby nuclear power plant at a rate of ~100 m^3^/s, and in an adjacent reference area with natural temperatures (Huss et al., 2019). This discharge varies between seasons but generally warms the Biotest Lake to 4–10℃ above the surrounding archipelago (Figure 1a), and created a paired temperature treatment for our mesocosms put in the heated enclosed coastal ecosystem and outside it in the reference area in the surrounding coastal area with normal temperatures.

The mesocosms consisted of sealed tube‐shaped plastic (polyethylene) bags with a volume of 4000 L (circular bottom with 1.6 m diameter, 2 m deep), attached to metal rings and floated from anchored rafts. To minimize the risk of bags breaking, we placed the experimental bags inside older and somewhat larger bags, with water also in between the bags. The 12 mesocosms (6 in the heated area and 6 in the reference area) were filled with filtered (70 µm mesh) sea water and inoculated with similar amounts of zooplankton from pooled samples collected in the surrounding waters (close to the mesocosms in the reference and heated area, respectively) on May 30. On May 31, we added 30 newly hatched perch larvae (15 from each of two egg strands, which were used for all mesocosms such that origin and initial size was identical across replicates) to each mesocosm (i.e., day 1 of the experiment). The egg strands were collected in the reference area on May 19 and kept in aerated indoor aquaria until hatching. Shortly before zooplankton addition, we browned half of the bags using 800 ml of Sera Blackwater Aquatan water conditioner (Sera GmbH, Heinsberg, Germany). Sera Blackwater makes the water brown and reduces light availability but has little influence on pH and nutrient levels (Van Dorst et al., 2020). This rendered four treatments: clear‐reference, clear‐warm, brown‐reference, and brown‐warm. We replicated each treatment three times and randomly assigned browning treatments within each of the two locations to the mesocosms. Four bags (one of each treatment) broke and/or took in water toward the end of the experiment and were therefore not included in the analyses.

### 2.2 Sampling and biological analyses

We took water samples for chlorophyll *a* (chl *a*) analyses at 1 m depth with a 0.6 L water sampler of which 500 ml was filtered onto 47‐mm‐dimeter glass microfiber filters (Whatman™), wrapped in aluminum foil and put in sealed bags that were stored frozen and later extracted with ethanol and measured with a spectrofluorometer (LS 30 PerkinElmer, Waltham, MA, USA) at Umeå Marine Sciences Center. We had temperature loggers in each area, taking hourly measurements during the duration of the experiment. Temperatures in the Biotest Lake (warm treatment) were on average 3.95°C warmer than in the reference area (reference treatment) (Figure 1a). We measured photosynthetically active radiation (PAR) at the surface, 0.5 and 1.5 m depth on days 6 and 13 with a LI‐250A light meter with a LI‐193SA spherical underwater quantum sensor (LI‐COR Biosciences, Biotechnology, Lincoln, NE, USA). We calculated light attenuation coefficients (*K_z_*) based on differences in PAR at the surface and depth *z* as: $K_{z}=\ln\left(\frac{{\mathrm{PAR}}_{0}}{{\mathrm{PAR}}_{z}}\right)/z$. *K_z_* was on average ~5 times higher in the brown (3.42) than in the clear (0.67) water treatments (Figure 1b). Sampling for zooplankton was done on days 1, 13, and 20 and for chl *a* on days 13 and 20 of the experiment (sampling for chl *a* was not done on day 1 due to a broken filtration unit). We sampled zooplankton by hauling a 70‐µm mesh net with a diameter of 25 cm from 1m depth and preserved the samples in Lugol's solution. Zooplankton were classified by taxa (cladocerans to genus level and copepods as either cyclopoid, calanoid, or nauplii) and up to 15 individuals per taxa and sample were length measured (all if fewer) using a stereo microscope and converted to biomass using length–weight regressions (Botrell et al., 1976, Dumont et al., 1975). Given a strong dominance of calanoid copepods across treatments (at the final day of the experiment: means of 97.8%–100% in the different treatments), we only report results on total zooplankton biomass. On the last day of the experiment (day 20), all mesocosms were sampled for fish with a large dip net, hauled from the bottom until five consecutive hauls resulted in zero fish captures. After capture, the fish were euthanized in a benzocaine solution and stored frozen until time of measurements. They were then blotted dry and measured (total length) and wet weight estimated to the nearest 0.1 mm and 0.0001 g. Note that given similar body sizes at the start of the experiment (all the larvae used originated from the same egg strands in all replicates), a difference in final body size is the same as a difference in body growth. For a subset of the captured fish (5 per mesocosm, all if fewer), we also estimated daily growth rates using otoliths. The sagittal otoliths were extracted, mounted on a microscope slide with Crystalbond epoxy, ground with fine sandpaper, and lightly polished until the sequence of fine lines assumed to be daily growth increments was visible, and then placed under a compound microscope (at 20 or 40 magnification, depending on size) attached to a camera and photographed. After combining images of different parts of the same fish otolith, we counted daily growth increments (present in all analyzed fish) from the core to the outer edge using the software ImageJ 1.52i (Rueden et al., 2017), with the distance between each count representing daily otolith growth rate.

### 2.3 Statistics

We analyzed treatment effects on final perch body length and survival with two‐way ANOVAs. Treatment differences in zooplankton biomass and chl *a* concentrations over time were analyzed with mixed‐design analyses of variance models (mixed ANOVA) with temperature and color as between‐mesocosm variables and date as a random within‐mesocosm variable using the package *afex* in R (Singmann et al., 2018). If we found significant interactive effects, we performed pairwise tests with Bonferroni adjustments using the *lsmeans* package in R (Lenth, 2016). Zooplankton biomass and chl *a* concentrations were ln‐transformed before analyses. We checked the assumption of sphericity for analyses with mixed ANOVA and assessed normality of residuals by visual examination of Q‐Q plots. All analyses were based on significance level *p* < .05 and done in R 3.4.3 (R Core Team, 2017).

## 3 RESULTS

**FIGURE 2:**
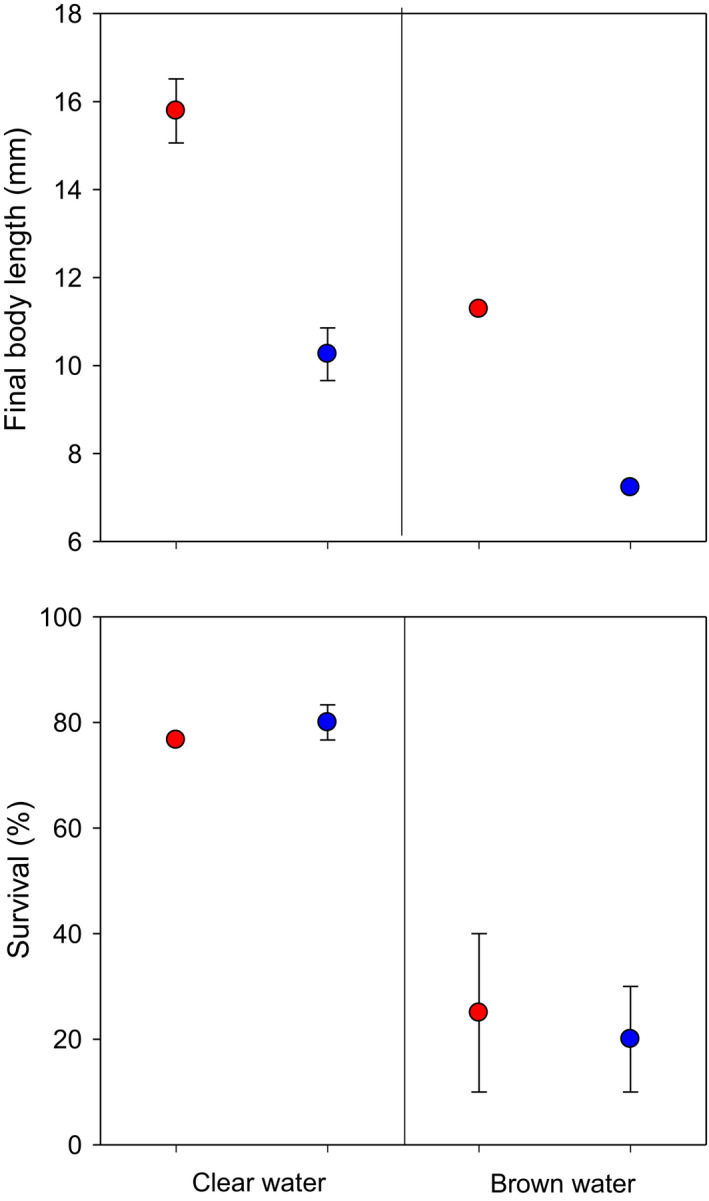
Final body length and survival (mean ± 1 SE) of perch in enclosures in the heated (red) and reference (blue) area with clear (left) or brown (right) water. Means are based on average length and survival in two mesocosms per treatment, in which 3–23 fish (out of 30 stocked, but note that only one bag in the brown‐reference treatment had surviving fish) were retrieved per mesocosm at the end of the experiment. See Table 1 for statistical results

**FIGURE 3:**
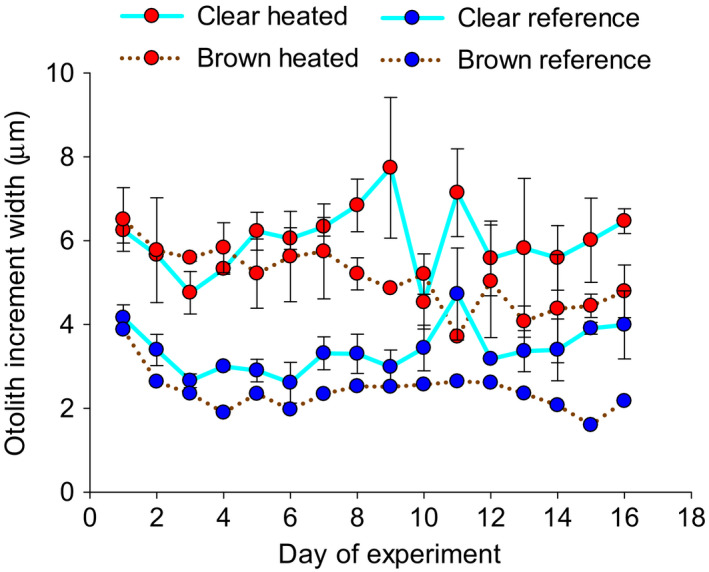
Daily otolith increment width (mean ± 1 SE, based on two mesocosms per treatment with 3–5 fish in each, except for one brown‐reference treatment where no fish survived) of larval perch in enclosures in the heated (red) and reference (blue) area with clear (solid turquoise lines) or brown (brown dotted lines) water

Perch body growth was higher in the heated compared with reference waters irrespective of water color. Growth was instead lower in brown waters irrespective of temperature, thus being highest in the clear‐heated and lowest in the brown‐reference treatment (Table 1, Figure 2). While the positive effect of heated waters manifested early in the growth history, the negative effect of browning on growth emerged first after ~1 week (compare daily otolith growth increments in Figure 3). Browning decreased perch survival in both reference and heated waters, whereas there was no effect of temperature on survival (Table 1, Figure 2).

**TABLE 1:** ANOVA models of the effects of water color and temperature on final perch body length (mm) and survival (proportion)

Explanatory variables	Body length	Survival
Water color	***F*_(1, 3)_ ** = 42.9**	***F*_(1, 3)_ ** = 44.0**
Temperature	***F*_(1, 3)_ ** = 52.9**	*F*_(1, 3)_ = 0.41
Water color × Temperature	*F*_(1, 3)_ = 1.47	*F*_(1, 3)_ = 1.00

Note

Significant results are in bold. *p ≤ .05; **p ≤ .01.

**FIGURE 4:**
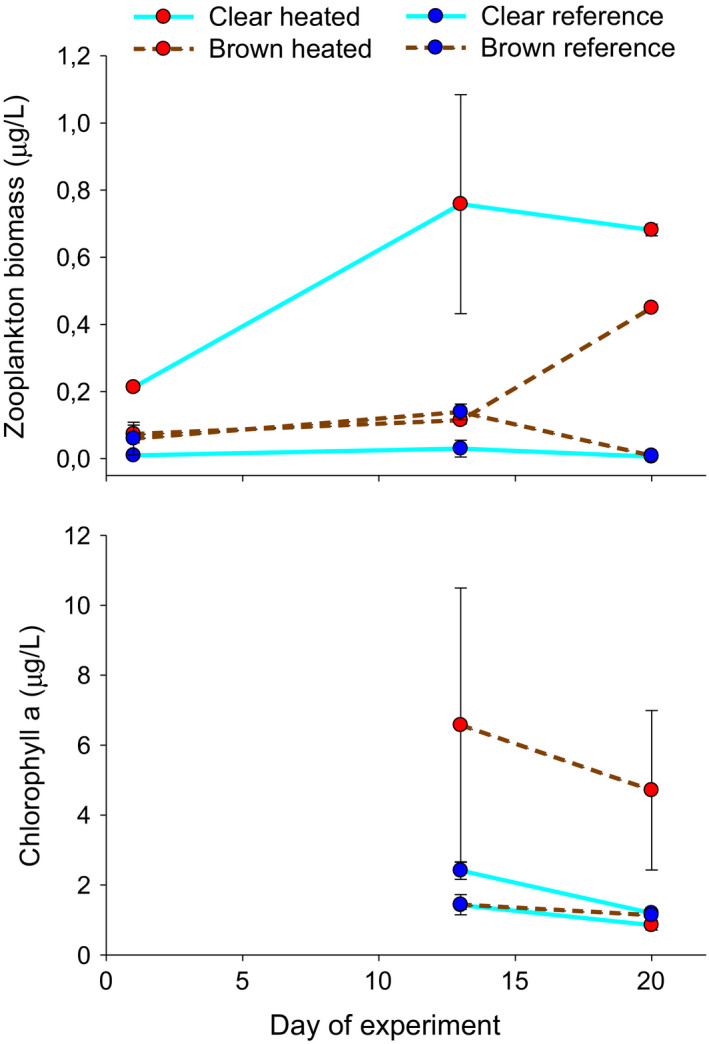
Zooplankton biomass and chlorophyll *a* (mean ± 1 SE) on three and two sampling dates, respectively, in enclosures in the heated (red) and reference (blue) area with clear (solid turquoise lines) and brown (brown dashed lines) water. See Table 2 for statistical results

Total zooplankton biomass was higher in heated than reference water treatments (Table 2). This was true irrespective of color treatment, although the difference developed earlier in clear than in brown water treatments (Table 2, Figure 4). Water color did not affect zooplankton biomass (Table 2). We found an interactive effect of water temperature and color on chl *a* levels (Table 2), with a positive effect of a high temperature only in brown water mesocosms (pairwise comparison *p* = .031, Figure 4). Copepods dominated the zooplankton community across treatments and time (overall mean = 92%), meaning no significant shifts in zooplankton community composition could be observed (results not shown).

**TABLE 2:** Mixed ANOVA models of the effects of water color, temperature, and time on total zooplankton biomass (µg/L) and chl *a* concentrations (µg/L)

Explanatory variables	Zooplankton biomass	Chl *a* concentration
Water color	*F*_(1, 3)_ = 0.10	*F*_(1, 4)_ = 3.42
Temperature	***F*_(1, 3)_** = 17.82*	*F*_(1, 4)_ = 1.92
Water color × Temperature	*F*_(1, 3)_ = 2.91	***F*_(1, 4)_** = 7.75*
Time	*F*_(2, 6)_ = 2.46	***F*_(1, 4)_** = 24.2**
Water color × Time	*F*_(2, 6)_ =v0.006	*F*_(1, 4)_ = 4.90
Temperature × Time	***F*_(2, 6)_** = 7.07*	*F*_(1, 4)_ = 0.17
Water color × Temperature × Time	*F*_(2, 6)_ = 1.16	*F*_(1, 4)_ = 0.34

Note

Significant results are in bold. * *p* ≤ .05; **p ≤ .01.

## 4 DISCUSSION

Ecosystems are rapidly changing due to global climate change and other anthropogenic pressures, but experimental research on organisms’ responses to such change commonly considers effects of only single drivers and ignores biotic interactions. Here, we experimentally show how warming and browning concurrently can influence the performance of larval fish. Whereas experimental browning decreased both body growth and survival of larval perch, warming increased body growth with no effect on survival. This contrasting effect of two environmental variables that both are increasing with climate change demonstrates the importance of accounting for multiple stressors to understand organism responses to climate change.

The higher body growth of small perch in warm waters confirms previous similar results from a long‐term study from the same heated ecosystem, showing a gradual but substantial increase in body growth of small (but not large) perch individuals following warming (Huss et al. (2019), see also Van Dorst et al. (2019) for a comparative lake study showing similar results). This suggests that the short‐term plastic responses observed here may be relevant also for long‐term responses. Increased body growth of juvenile individuals is also in line with the so‐called temperature‐size rule, suggesting that warming should result in faster growth and development, but smaller adult size, which is observed across many taxa (Atkinson, 1994; Ohlberger, 2013). Both maintenance costs and feeding rates scale strongly, but differently, with temperature in fish (Lindmark 2020) as in other ectotherms (Gillooly et al., 2001, Englund et al., 2011), with surplus energy available for growth being maximized at some intermediate optimum temperature. Positive growth responses to warming are expected when the temperature increase results in closer to optimum temperatures for body growth, which for many fish species is much higher for small than for large individuals (Huss et al., 2019; Imsland et al., 2006; Lindmark 2020; Pörtner & Farrell, 2008). Accordingly, the larval perch in our experiment could exhibit higher growth rates in the heated water. This higher body growth of larval perch in warm water treatments likely had little to do with food availability. Daily growth increments (Figure 3) showed that the difference in body growth between temperature treatments was initiated already from the start of the experiment, irrespective of water color and despite initially similar larval body sizes (and larvae of identical origin) and zooplankton levels (Figure 4). This suggests that the warming‐induced differences in body growth largely developed because of direct physiological responses to warming rather than through temperature‐induced shifts in prey availability. These findings also highlight the benefit of looking at detailed growth patterns to understand how and why environmental factors affect early life history of fish.

Although growth patterns suggest that warming effects on larval fish performance were only weakly linked to zooplankton availability, the zooplankton themselves did respond to warming as well. Despite high fish survival and body growth in warm treatments, indicating high consumption rates on zooplankton, warming still increased zooplankton biomass. The higher zooplankton biomass in warm waters did, however, not lead to a decrease in chlorophyll a concentrations. Instead, in the brown treatment, we observed a positive effect of warming on chlorophyll. Previous studies suggest that warming effects on phytoplankton vary (but are often negative) with nutrient supply, associated shifts in cell size, grazing pressure, and other factors (Bernhardt et al., 2018; Schulhof et al., 2019; Uszko et al., 2017). The contrasting lack of a positive effect on chlorophyll concentrations in the clear water treatments, where the highest zooplankton biomass was observed, suggests that zooplankton may remove potential warming effects on phytoplankton, unless limited by dark environments.

Lower perch body growth in brown compared to clear water treatments irrespective of temperature may result from lower availability of preferred prey and/or lower feeding rates because of reduced light penetration. The lack of a browning effect on zooplankton community biomass or composition contrasts to some earlier studies (Leech et al., 2021; Robidoux et al., 2015), including one using a similar level of browning (van Dorst et al., 2020). The likely explanation is that such an effect depends on the initial zooplankton composition, which in this study was dominated by copepods (on average constituting 92% of the total zooplankton biomass). The lack of a browning effect on copepods is in line with findings in van Dorst et al. (2020), suggesting that zooplankton communities dominated by copepods, such as commonly found at sea and some coastal areas, may be less susceptible to browning‐induced changes than freshwater communities that more often are dominated by cladoceran zooplankton. The fact that browning did not affect zooplankton biomass suggests that slower body growth rates of fish larvae in this experiment mainly came about because of light limitation reducing their feeding rates rather than lack of prey. This is in line with earlier findings showing that browning and DOC can influence foraging rates of some, but not all, zooplanktivorous fishes (Van Dorst et al., 2020; Weidel et al., 2017). That reduced foraging efficiency plays a role here is also supported by the fact that survival was lower in brown water treatments, irrespective of temperature and zooplankton biomass. This indicates that larval perch need higher concentrations of zooplankton to survive in brown waters than in clear waters, because of their less efficient feeding in the darker environment. In contrast to our findings, a freshwater mesocosm experiment of browning effects on perch by van Dorst et al. (2020) suggested that it mainly was a change in prey composition, and visibility only to a lesser extent, that negatively affected perch body growth. However, it is possible that negative effects of decreased visibility manifest mainly at very early life stages, as in our study. There are few previous studies on larval fish responses to browning to use for comparison (Leech et al., 2021 with larval largemouth bass and bluegill), and even fewer outside the laboratory in combination with other factors, such as temperature (but see Hansson et al. (2013) for a tank experiment with roach and treatments varying humic matter and temperature), making it difficult to generalize about impacts of brown water on larval fish due to reduced foraging rates. Another potentially contributing factor (not studied here) for lower body growth and survival could be reduced zooplankton nutritional quality in brown water treatments, in turn, for example, linked to lower concentrations of essential fatty acids in phytoplankton in brown waters (Taipale et al., 2018). The latter may, however, be more important in the case of natural browning including associated carbon and nutrients than in cases with only a light effect, as in our study. Finally, it should be noted that more replication and frequent measurements of the prey community could have increased chances of finding any minor influence of prey availability on larval fish performance.

Resolving how climate change affects food webs requires accounting for the multiple ways in which it is altering natural environments (Boyd et al., 2018), as well as scaling up responses of individual organisms to populations and food webs. During recent decades, lakes and many coastal waters across the northern hemisphere have become both warmer and browner (Creed et al., 2018). Similar to comparative lake studies (shown for trout in Symons et al., 2019 and for juvenile but not adult perch in van Dorst et al., 2019), we found that such higher temperatures and darker waters may have antagonistic effects on fish body growth. This suggest that the widespread notion of a temperature‐size‐rule, predicted to result in increased growth rates of juvenile fish (Atkinson, 1994), may not be general but context dependent—as positive physiological effects of warming can be negated by lower foraging efficiency in brown environments. Similarly, the expected negative effects of browning may not be visible in fish if they are simultaneously exhibiting warming waters. Whereas warming is a global phenomenon, there is great spatial variation in the extent to which waters are getting browner (Kritzberg et al., 2020). Thus, we can expect different larval fish growth and survival responses to a changing climate depending on the geographical context. The 3.95℃ higher than reference temperature measured in the heated enclosures during our experiment is higher than the extent of natural warming observed but is still within the range predicted for surface temperatures until 2100 for this region (with a higher than average increase expected for the Baltic Sea, IPCC, 2014).

Scaling up responses for a specific life stage, such as observed in our experiment, to that of whole populations is difficult. Whereas warming may mitigate the negative impacts of browning on larval perch body growth (as we found), there is strong evidence that high temperatures will negatively affect whole perch populations, adding to the negative effects of browning (Van Dorst et al., 2019). Lower population production in warm environments comes about despite no or even positive effects on body growth in small life stages (Huss et al., 2019; Van Dorst et al., 2019) due to a lower proportion of large individuals (van Dorst et al., 2019). Thus, while warming and browning can have different effects on individual body growth of fish larvae, and their effects might even cancel each other out, their effects on overall population growth can be additive. Experimental tests of such population‐level responses to multiple pressures are however lacking for fish.

In conclusion, experimental warming and browning have contrasting effects on larval perch performance, where physiological responses likely explain the faster body growth in warm waters and reduced foraging efficiency explains the slower body growth and higher mortality in brown waters. We conclude that browning of waters may remove any positive effect of warming on perch larval performance, and warming may remove any negative effect of browning. Our results highlight the importance of examining the combined impact of climate stressors, exemplified by that fish body growth responses to future climate change may depend on the relative rate and extent of both warming and browning.

## CONFLICT OF INTEREST

The authors declare no conflicts of interest.

## AUTHOR CONTRIBUTION

**Magnus Huss:** Conceptualization (equal); Formal analysis (lead); Funding acquisition (equal); Methodology (equal); Writing‐original draft (lead). **Renee van Dorst:** Conceptualization (equal); Formal analysis (supporting); Methodology (equal); Writing‐review & editing (supporting). **Anna Gardmark:** Conceptualization (equal); Formal analysis (supporting); Funding acquisition (equal); Methodology (equal); Writing‐review & editing (supporting).

## Data Availability

The data used for this manuscript are uploaded to Dryad: https://doi.org/10.5061/dryad.n02v6wwz5
